# A case for limited global contraction of Mercury

**DOI:** 10.1038/s43247-020-00076-5

**Published:** 2021-01-14

**Authors:** Thomas R. Watters

**Affiliations:** Center for Earth and Planetary Studies, National Air and Space Museum, Smithsonian Institution, Washington, DC 20560-0315 USA

**Keywords:** Tectonics, Tectonics

## Abstract

Mercury is a one-plate planet that has experienced significant radial contraction primarily driven by interior cooling. In some previous studies aimed at estimating the total magnitude of contraction, numerous faults are assigned to positive relief landforms, many without evidence of origin by deformation, resulting in estimates of planetary radius reduction as large as 7 km. Here we use high-incidence angle image mosaics and topography from the MESSENGER mission to map Mercury’s contractional landforms. Each landform is assigned a single, principal fault, resulting in an amount of contractional strain equivalent to a radius change of no more than 1 to 2 km. A small radius change since the end of heavy bombardment is consistent with Mercury’s long-lived magnetic field and evidence of recent tectonic activity. It is concluded that the retention of interior heat and a lower degree of contraction may be facilitated by the insulating effect of a thick megaregolith.

## Abstract (web summary)

Planet Mercury’s radius probably reduced by no more than 2 km since the Late Heavy Bombardment, with a strong hemispheric dichotomy in contractional strain, suggesting the loss of interior heat over time is less than expected for a small rocky body

## Introduction

Mercury is a planet dominated by contractional deformation and could be considered the archetype in our solar system of how a one-plate planet expresses the loss of its interior heat. The preserved, post-late heavy bombardment (LHB) tectonic history of Mercury is so dominated by contraction that the occurrence of extensional landforms (i.e., graben) is restricted to the interior volcanic plains of impact basins^1–4^ and some volcanic plains that buried craters (ghost craters) and basins^5–7^. The first hint of this dominance was revealed in the hemisphere imaged by Mariner 10^8–10^. Among the most remarkable discoveries that can be attributed to the MErcury Surface, Space ENvironment, GEochemistry, and Ranging (MESSENGER) mission during three flybys and over four years in orbit is the detection of hundreds of large-scale tectonic landforms indicative of planetary contraction. Flyby and orbital imaging by the Mercury Dual Imaging System (MDIS) wide-angle and narrow-angle cameras^11^ provided targeted images and global image mosaics. Early in the orbital phase of the mission, after the first solar day, it was recognized that the low-incidence to moderate-incidence angle monochrome images that had been acquired were not optimum for the identification of tectonic landforms. An imaging campaign was then initiated to obtain high-incidence angle image coverage. This resulted in two near-global high-incidence angle (65° to 88°) mosaics with opposite, east and west, solar azimuth directions with pixel scales of ~166 m. During the last 18 months of the MESSENGER mission, the spacecraft’s periapsis altitude was lowered, providing the opportunity to image the surface at much higher spatial resolution (see ref. ^12^). The images and mosaics combined with topography from the Mercury Laser Altimeter (MLA)^13^, stereo imaging^14,15^, and image-based control-point network techniques^16^ have facilitated the production of regional and global maps for use in identifying and mapping tectonic landforms.

**Fig. 1:**
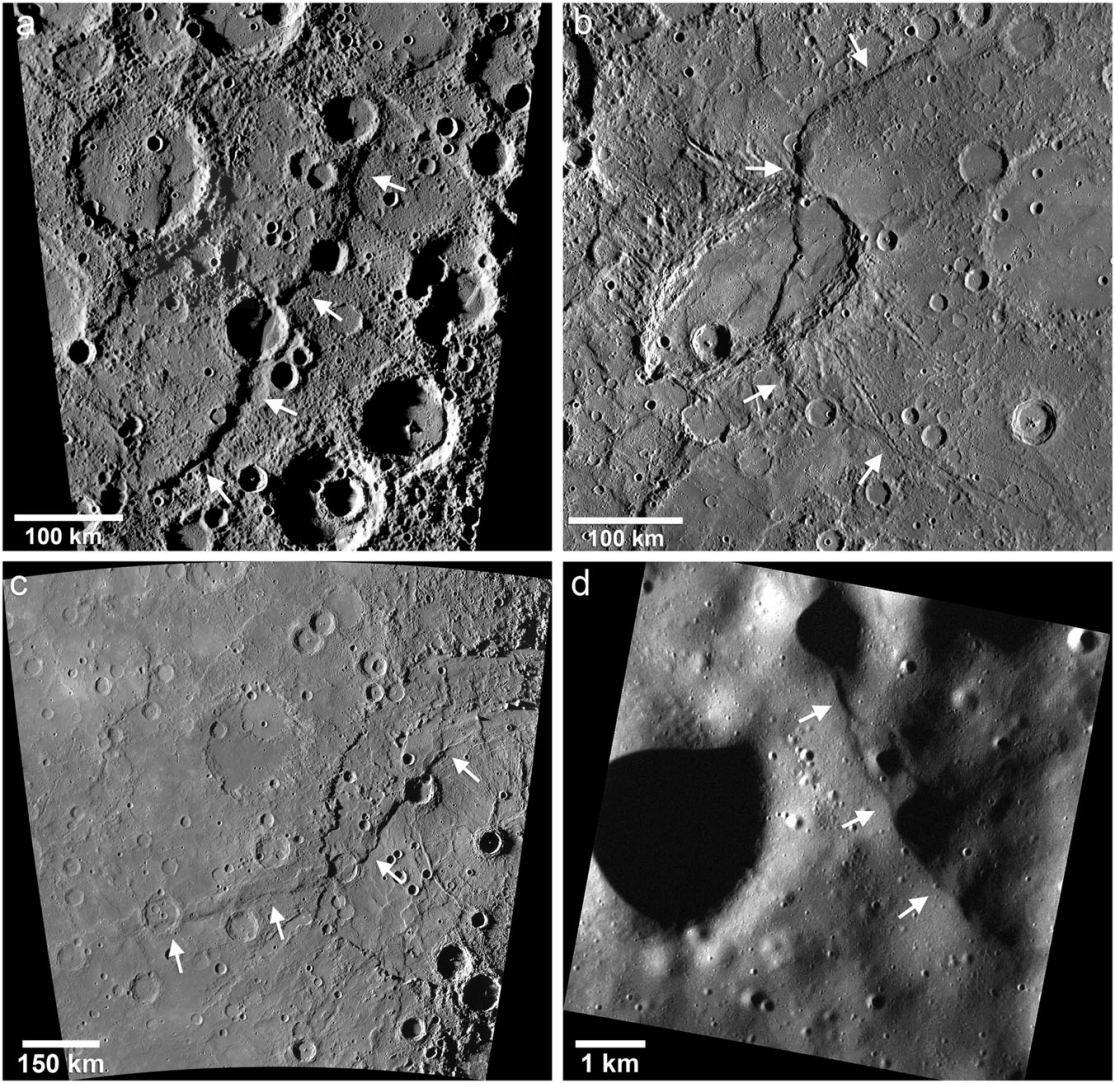
Mercury’s lobate thrust fault scarps. **a** MDIS high-incidence angle image mosaic of Discovery Rupes (~55°S, 37°W), one of the prominent lobate scarps revealed during the Mariner 10 mission. **b** The bow-shaped Beagle Rupes (~2°S, 101°E) crosscuts the elliptically shaped Sveinsdóttir crater. Beagle Rupes was revealed during MESSENGER’s first flyby. **c** Enterprise Rupes (~36°S, 77°E), the largest lobate scarp on Mercury, was revealed during MESSENGER’s second flyby. This scarp cuts across the rim and floor of the Rembrandt basin, and forms the northern flank of Mercury’s great valley^20^. **d** Young, small-scale scarp (~38.9°N, 27.9°E) detected in the low altitude phase of the MESSENGER mission^12^. These small lobate scarps comparable in size and morphology to small lobate fault scarps found on the Moon. Image frame number EN1044893953M. White arrows indicate the locations of the fault scarps.

**Fig. 2:**
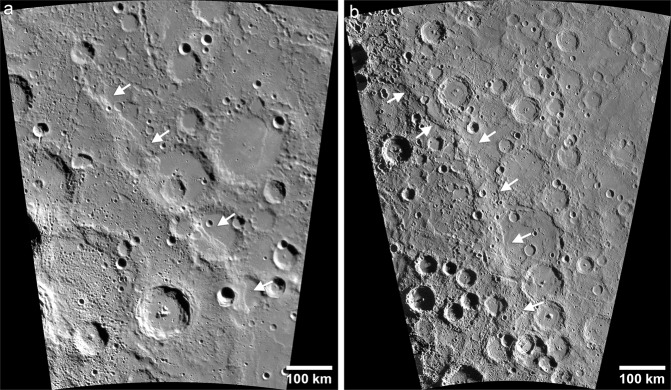
Mercury’s high-relief ridges. **a** MDIS high-incidence angle image mosaic of a prominent high-relief ridge (~66°S, 50°W) first revealed in the hemisphere imaged by Mariner 10^22^. White arrows indicate the location of the ridge. **b** A high-relief ridge with a maximum relief of over 2000 m (~58°S, 105°E) revealed during MESSENGER’s first fly (left-facing arrows)^17,18^. The ridge transition into a lobate scarp (right-facing arrows) near its northern terminus.

**Fig. 3:**
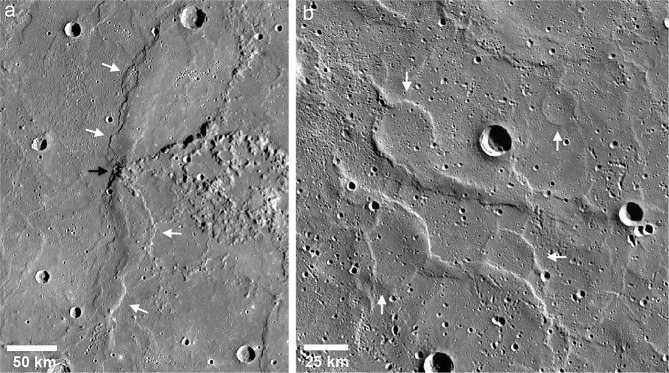
Mercury’s wrinkle ridges. **a** Schiaparelli Dorsum is a prominent wrinkle ridge (~23°N, 164°W) in the smooth plains of Odin Planitia. One of the largest wrinkle ridges on Mercury, Schiaparelli exhibits the classic morphologic elements of planetary wrinkle ridges, a broad arch and superpose ridge (white arrows). Note the undeformed appearance of the lens of hummocky terrain embayed by smooth plains volcanics and transected by the ridge (black arrow). **b** MDIS moderate-incidence angle mosaic of wrinkle ridges in the northern smooth plains. Wrinkle ridge rings or ghost craters (white arrows) are common in the northern smooth plains (~79°N, 93°E) and other smooth plains units on Mercury^5,27^. Ghost craters express the influence of shallow buried impact craters in the localization of wrinkle ridges.

Although the Mariner 10 and MESSENGER flyby images revealed many tectonic landforms^17,18^, the complete picture of the variety and scale of Mercury’s contractional features did not emerge until orbital images were returned^5,12,19,20^. Mercury’s tectonic landforms can be divided into two spatially distinct classes, broadly-distributed and basin-localized. Broadly distributed contractional tectonic landforms on Mercury fall into three distinct and well characterized landforms; lobate scarps (Fig. 1), high-relief ridges (Fig. 2), and wrinkle ridges (Fig. 3). Lobate scarps are by far the most broadly distributed and have the greatest range in scale, with over 3 km of relief on the largest scarp^20^ and only tens of meters of relief on the smallest^12^. They are clearly distinguishable from the other contractional landforms by their asymmetric cross-section, consisting of a steeply sloping scarp face and a gently sloping back limb (Fig. 1). Vertical offset of crosscut impact crater walls and floors are interpreted to be evidence of surface-breaking thrust faults^8–10,21–23^. Lobate thrust fault scarps occur predominantly in intercrater plains, began forming after the end of LHB^24^, and are likely still forming today^12^. High-relief ridges are closely related to lobate scarps but are morphologically distinct and less common, characterized by a more symmetric form and generally no clear expression of surface breaking faults^17,18,22^ (Fig. 2). Based on their distribution, lobate scarps and high-relief ridges result from global contraction due to interior cooling with some contribution of stresses from other global-scale sources (see ref. ^19^).

**Fig. 4:**
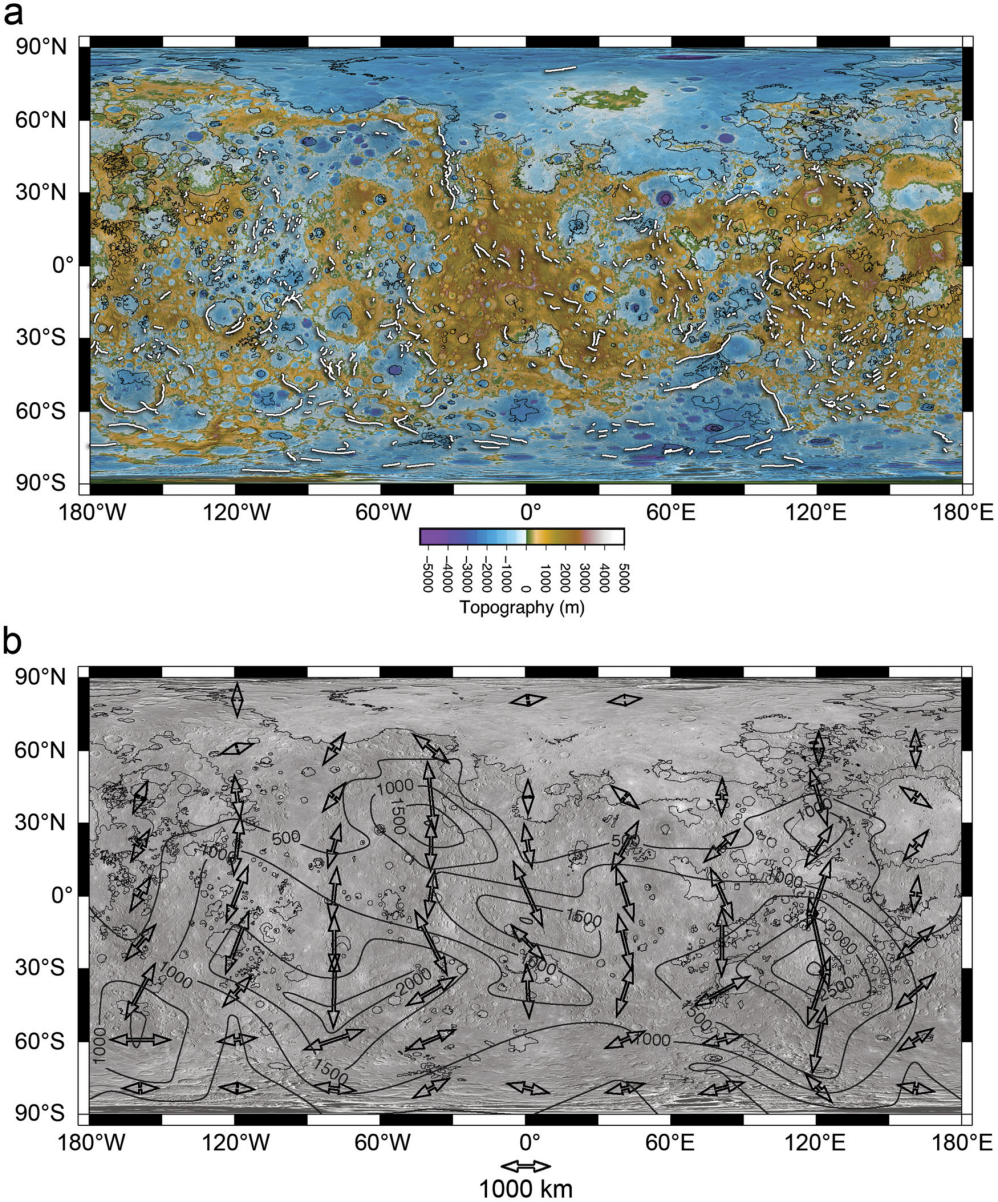
Global distribution of lobate thrust fault scarps and high-relief ridges on Mercury. **a** The locations of lobate scarps and high-relief ridges (white polylines) are shown on the topographic map of Mercury^16^. **b** Plot of orientation vectors and fault density. Orientation vectors are the median orientations of mapped lobate scarp and high-relief ridge segments scaled by cumulative length (in km) of segments in sample areas with dimensions of 40° × 20° (longitude and latitude). The contours are lines of equal fault density expressed by the cumulative length (in kilometers) of mapped structures within the sample areas. The contour interval is 500 m. Smooth plains units^26^ are outlined (thin black lines) in **a** and **b**.

The largest fault scarp on Mercury, Enterprise Rupes, has the greatest relief, is ~1000 km long, and crosscuts the rim and floor of the Rembrandt basin (Fig. 1c). A second scarp complex to the south, Belgica Rupes, extends to the rim of Rembrandt. These large scarps have opposite-facing vergence and border a relatively flat-floored valley with a mean width of ~400 km (~40°S, 80°E) (Fig. 4a)^20^. The valley floor is significantly offset below the elevation of the back-scarp terrains^20^.

Smooth volcanic plains cover ~27% of Mercury’s surface (Fig. 4)^25–27^. The largest expanses of smooth plains are found in the northern hemisphere with ~23% of the total in the northern hemisphere and only ~4% of the total in the southern hemisphere (Fig. 4b). This dichotomy in the distribution of volcanic plains is very reminiscent of the Moon. The total percent of the lunar surface covered by mare basalt is ~16% with ~15% of the total mare basalts on the nearside and only ~1% on the farside^28^. Smooth plains are dominated by wrinkle ridges forming expanses of ridged plains, including those in the large impact basins Caloris and Rembrandt. Ghost craters, wrinkle ridge rings localized by shallow buried impact craters, are common in smooth plains^27^ (Fig. 3b). Wrinkle ridges on Mercury typically have hundreds of meters of relief, generally greater than their counterparts on the Moon^29,30^ and Mars^29^. Mercury’s wrinkle ridges, like wrinkle ridges in lunar mare basalts, likely formed by stresses resulting from load-induced subsidence and lithospheric flexure in response to emplacement of thick, basalt-like volcanic sequences with some contribution from global contraction^18,30–32^.

Mercury’s contractional tectonic landforms have been used to estimate the amount of post-LHB radius change, and these estimates vary greatly. Estimates have been based on regional surveys of: (1) the hemisphere imaged by Mariner 10^8,21^, (2) areas imaged during MESSENGER flybys^33^, (3) combination of areas imaged during Mariner 10 and MESSENGER flybys^18^, and (4) estimates from global surveys of the surface imaged during MESSENGER’s orbital phase^34,35^. In these works, regional estimates of contractional strain are extrapolated on the assumption they are representative of the entire surface, while global estimates are based on the assumption that contractional strain is homogeneously distributed.

Mariner 10-era estimates of radius change are about 1 to 2 km^8^ with most values near ~1 km from estimates of the global contractional strain^21,31^. A super-contracted Mercury (defined as radius change »2 km) has been suggested in the MESSENGER-era. A radius change as large as ~4 km^33^ to ~7 km^34,35^ has been reported, with even larger amounts due to putative unexpressed contractional strain^36^. These much larger estimates of radius change are a direct result of the approach taken in the interpretation and mapping of the contractional tectonic landforms (Supplementary Note 1, 2). Lobate scarps, high-relief ridges, wrinkle ridges, and many positive relief landforms lacking definitive evidence of origin by deformation are often grouped together as shortening structures^34,35,37,38^. In some studies^34,35,37,38^, it is assumed that contractional landforms cannot be separated or described using the traditional classification based on morphometry, complexity, and geologic setting, leading to the misinterpretation of many positive relief features as tectonic in origin (Supplementary Note 2, Supplementary Figs. 1, 2). Individual contractional landforms are often assigned multiple, primary faults. This leads to problems accounting for the implied subsurface geometry of numerous faults in close proximity and the kinematics of formation. Large overestimates are expected when; (1) single structures are represented by multiple faults, (2) features lacking evidence of origin by deformation are assigned faults, and 3) all faults are weighted equally in the assessments of contractional strain^34,35^ (Supplementary Note 2, Supplementary Figs. 1, 2).

## Results

### Spatial distribution of faults by hemisphere

The approach taken in the interpretation and mapping of the tectonic landforms is critical to the analysis of the spatial distribution and to estimates of the contractional strain. Here, each lobate scarp and high-relief ridge is interpreted to have a single primary thrust fault controlling the surface expression and contractional strain of the structure, and thus each is mapped with a single polyline (see Supplementary Note 1). The new tectonic map, generated using high-incidence angle mosaics and global topographic data not available in a previous survey of only the largest contractional landforms^19^, shows the spatial distribution and areal density of all the identified contractional landforms is not uniform (Fig. 4a). This is consistent with the previous analysis of the most prominent lobate scarps and high-relief ridges^19^. Orientation vectors, median orientations scaled by total fault length within sample areas (40° longitude × 20° latitude), illustrate that there are areas where the cumulative length of faults is greater (Fig. 4b). The largest concentration of lobate scarps and high-relief ridges is centered at roughly 30°S, 120°E, east of the Rembrandt basin (Fig. 4b). This analysis clearly shows a dichotomy in cumulative length of mapped faults between the northern and southern hemispheres. For example, there are three regions in the southern hemisphere with cumulative fault lengths greater than 1500 km, compared to only one region in the northern hemisphere (centered ~30°N, 40°W). Total length of faults in the southern hemisphere (~43,260 km) is more than a factor of two greater than the total length in the northern hemisphere (~18,430 km). This hemispheric dichotomy in cumulative length clearly indicates the spatial distribution of contractional strain is not uniform. The difference between the two hemispheres cannot be accounted for by illumination bias (see ref. ^19^), or burial of preexisting contractional landforms by smooth plains volcanism. The stratigraphic relation between lobate scarps and impact craters suggests that thrust faulting started during, or not long before, the Calorian, the period when smooth plains volcanism occurred^24^. Thus, a significant portion of the population of lobate scarps would not be expected to have been buried by the smooth plains of the northern hemisphere.

The distribution of the longest faults also contribute to the difference in total length between the hemispheres, with 74% of all mapped faults >100 km in length occurring in the southern hemisphere. The areal distribution of the wrinkle ridge dominated smooth plains in the northern hemisphere contributes to this hemispheric dichotomy, however, there are also expanses of intercrater plains in the northern hemisphere with relatively few contractional tectonic landforms (Fig. 4a, b). None of the current models proposed for the origin of the stress^19^ accounts for the observed hemispheric dichotomy in contractional strain.

### Displacement-length relations

The population of contractional landforms has been used to estimate the amount of post-LHB radius change experienced by Mercury. Key to an accurate estimate of the contractional strain and corresponding radius change is the characterization of the displacement-length (*D/L*) ratio γ of the thrust faults associated with the population of lobate scarps and high-relief ridges (see Supplementary Note 3). From the measured maximum relief of the scarp *h*_max_, the maximum displacement is given by *D*_max_ = *h*_max_/*sin* *θ* for a range of expected dips θ for thrust faults of 25° to 35° ^39^. This range is consistent with fault dips of the Wind River thrust fault and thrust faults in the Rocky Mountain foreland of Wyoming, terrestrial analogs to lobate thrust fault scarps (see ref. ^40^).

**Fig. 5:**
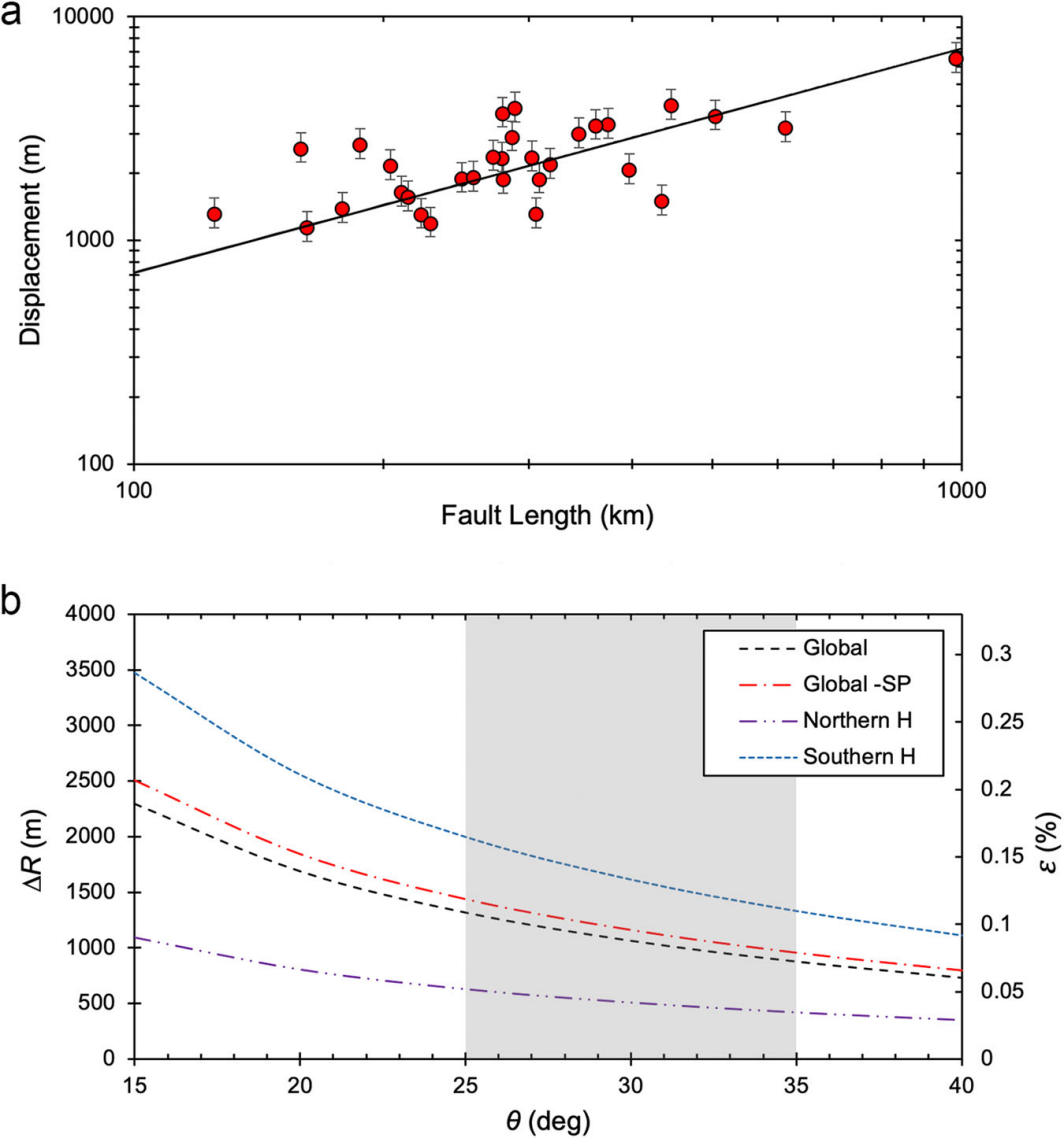
Displacement-length relations and radius change. **a** Log-log plot of maximum fault displacement as a function of fault length for 31 lobate scarps (red circles) on Mercury. The ratio γ of the population of thrust faults was obtained by a linear fit to the *D–L* data with the intercept set to the origin. A value of *γ* ≅ 7.2 × 10^3^ is obtained using estimates of *D*_*max*_ based on fault plane dips *θ* = 30°. The error bars indicate the range in displacement for *θ* = 25°, 35°. **b** Plot of radius decrease Δ*R* and areal contractional strain ε as a function of fault plane dip *θ*. Δ*R* and ε are determined for four cases; (1) all mapped faults over the total surface area of Mercury (black dashed line), (2) the total surface area less the smooth plains volcanic units and mapped faults within (red dashed single-dot line), (3) the surface area of the southern hemisphere and mapped faults within (blue short-dashed line), and (4) the surface area of the northern hemisphere and mapped faults within (purple dashed double-dot line). Over the likely range of *θ* of typical thrust faults (25° to 35°, area shaded in gray), Δ*R* only reaches ~2 km in the southern hemisphere case.

The maximum relief of a representative sample of the population of lobate scarps (*n* = 31) with a wide range in length scale was measured using the best available topographic data (see Supplementary Note 3, Supplementary Table 1) to estimate the maximum displacement on their respective thrust faults. The range of *D*_max_ is estimated to be ~1.1 to 6.5 km, assuming fault plane dips of 30°, with an average of ~2.4 km. The values of *γ* for *θ* = 25°, 30°, and 35° are determined by linear fits to *D*_max_/*L* data for the measured lobate scarps (Fig. 5a). Values of *γ* range from (~6.3–8.6) × 10^−3^ with *γ* ≅ 7.2 × 10^−3^ for θ = 30°. For the range in θ, the standard deviation of the slope *γ* is (~0.36–0.49) × 10^−3^.

### Planetary radius change globally and by hemisphere

Modeling of lobate scarp thrust faults indicates that they are deeply rooted, likely penetrating the entire mechanical lithosphere^20,23,41–43^. Thus, lobate scarps are the most direct tectonic indicators of lithospheric contractional strain. The areal contractional strain for large faults (*L* ≥ the maximum depth of faulting) is given by

1 $$\varepsilon = \frac{{{\mathrm{cos(}}\theta {\mathrm{)}}}}{A}\mathop {\sum }\limits_{k = 1}^n D_kL_k$$

where *θ* is the fault plane dip, *A* is the survey area, and *n* is the total number of faults^44^. Substituting *D* = *γL* in equ. 1 yields

2 $$\varepsilon = \frac{{\gamma \,{\mathrm{cos(}}\theta {\mathrm{)}}}}{A}\mathop {\sum }\limits_{k = 1}^n L_k^2$$

allowing the areal strain to be estimated from the sum of the squares of the lengths of the faults in the population. Using the range of *γ* given in the previous section and the total length of the mapped lobate scarp and high-relief ridge faults over the entire surface of the planet (~61,690 km), the global contractional strain is estimated to range from ~0.072% to ~0.11% (~0.09% for θ = 30°).

The radius change due to global contraction can be expressed by Δ*R* = *R*_*d*_ – R_*u*_ where *R*_*u*_ is the pre-deformation planetary radius and *R*_*d*_ is the post-deformation (current) planetary radius. *R*_*u*_ is related to the contractional strain by

3 $$R_u = \left[ {\frac{{R^2_d}}{{\varepsilon + 1}}} \right]^{0.5}$$

(see ref. ^31^). The radius decrease corresponding to the global areal contractional strain for the range in θ (25° to 35°) is ~0.9–1.3 km (~1.1 km for *θ* = 30°) (Fig. 5b).

An estimate of the global contractional strain can also be obtained by eliminating the area covered by the wrinkle ridge dominated smooth plains. In contrast to other MESSENGER-era studies^33,34^, wrinkle ridges are not included in the estimate of global contraction because the contribution of stresses from load-induced subsidence and flexure cannot be separated from that of global stresses^18,30,32^. The significant contribution of localized stress in wrinkle ridge formation is supported by ridge orientations that are often strongly influenced by the boundary conditions of the smooth plains they deform^10,31^. Ghost craters are compelling evidence that wrinkle ridge thrust faults are shallow rooted and that deformation is largely confined to flood volcanic sequences. In the northern smooth plains, covering more than 6% of the surface of Mercury, ghost craters are ubiquitous and reach diameters >100 km^27^ (Fig. 3b). Ghost craters are likewise common in volcanic plains on Mars and in mare basalts on the Moon.

The areal contractional strain excluding the smooth plains and lobate scarps and high-relief within (total length of faults ~52,954 km) is estimated to be ~0.08–0.12% (~0.1% for *θ* = 30°). This range in the contractional strain corresponds to a radius decrease of ~1.0–1.4 km (~1.2 km for *θ* = 30°) (Fig. 5b). Because of the hemispheric dichotomy in cumulative length and scale of the contractional faults, the lithospheric contractional strain is also evaluated by hemisphere. Estimates based on the contractional strain in the southern hemisphere establish an upper limit on the post-LHB radius change because the area covered by smooth plains is a minimum, the contractional features are more uniformly distributed, and their cumulative length is the greatest (Fig. 4b). The areal contractional strain based on the lobate scarps and high-relief in the southern hemisphere alone is estimated to be ~0.11–0.17% (~0.13% for *θ* = 30°) corresponding to a radius decrease of ~1.3–2.0 km (~1.6 km for *θ* = 30°) (Fig. 5b). The contrast in the contractional strain and radius change obtained for the northern and southern hemispheres is striking. In the northern hemisphere, the contractional strain is estimated to be ~0.03–0.05% (~0.04% for *θ* = 30°), corresponding to a radius decrease of ~0.4–0.6 km (~0.5 km for *θ* = 30°) (Fig. 5b). These estimates include lobate scarps and high-relief ridges mapped within the smooth plains. The small-scale scarps detected in the low altitude phase of the MESSENGER mission are not included in the estimates of radius change because their contribution to the contrational strains is not significant. It should be noted that other unaccounted for sources such as secondary accommodation faults or other forms of distributed or elastic strain are also not expected to significantly contribute to the contractional strain. The possible contribution from these other sources is likely captured by the estimated contractional strain over the range of *θ* assumed for primary thrust faults.

## Discussion

A super-contracted Mercury (radius change »2 km) is not supported by the population of mapped thrust faults in the intercrated plains with clear expression at the surface even when the radius change is based on the contractional strain in the southern hemisphere alone. For the radius change to greatly exceed 2 km, the contractional strain reported in this study must be significantly larger or unrealistically low values of θ must be invoked (Fig. 5b). Lower fault dip angles (*θ* < 25°) are not supported by terrestrial analogs and forward mechanical modeling of lobate scarps on Mercury (see Supplementary Note 3).

The motivation to find large amounts of post-LHB radius change has been fueled by predictions based on thermal history models for Mercury. The early thermal models^45–48^ are strongly dependent on assumptions about the size of Mercury’s inner core. It has been estimated that complete solidification of Mercury’s core would lead to a reduction in planetary radius of about 17 km^45^. Other thermal models have examined the effect of adding sulfur to the core chemistry, as well as how sulfur depresses the Fe–S eutectic and prolongs the liquid phase of the core^49,50^. However, a core with a high bulk sulfur content of >6.5 wt%, an amount that could account for 1 to 2 km of radial contraction, has been considered unrealistic^50^.

A more recent thermal model that predicts ongoing contraction requires a thin mantle (300–500 km), volcanic activity and secondary crust generation that lasts beyond LHB, and large amounts of net global contraction (≥3 km of radius change), invokes late-stage freezing of the inner core to generate a current magnetic field^51^. However, the presence of a global magnetic field and the discovery of a 3.9 Ga remnant magnetic field preserved in smooth plains volcanic rock^52^ poses a significant challenge to this and other such thermal history models. The discovery of small lobate scarps, on a scale with very young lobate thrust fault scarps found on the Moon^53–55^, suggests recent and likely ongoing tectonic activity on Mercury^12^. Very recent or current tectonic activity on Mercury is consistent with a dynamo sustained, at least in part, by prolonged, slow cooling of the interior. Thus, thermal models must now account for the slow cooling of Mercury’s interior, a sustained core dynamo, recent tectonic activity, and a total amount of post-LHB global contraction of ≤2 km.

Recent experimental work to investigate the insulating properties of core materials shows that a liquid FeS layer overlying a silicon-bearing core has an insulating effect, inhibiting freezing of Mercury’s inner core^56^. A layer rich in FeS greater than 40 km thick can sustain high temperatures across the core. Such a condition could result in a small or absent solid inner core, greatly restricting the amount of radius change^56^.

Another potentially important factor in preserving interior heat is the insulating effect of a thick megaregolith^57,58^. Like the Moon^59^, Mercury probably has a highly fractured, thick megaregolith. The insulating effect of a fractured, porous, low conductivity megaregolith has been proposed as a mechanism to inhibit interior cooling of the Moon^57^. A thick lunar megaregolith could eliminate the need to invoke a cold central interior immediately after the Moon’s accretion^57^ to account for the small amount of radial contraction expressed by lunar lobate scarps^53,54^. The modest amount of radial contraction of Mercury since the end of LHB reported here points to an evolutionary path for one-plate planets where interior heat is retained and tectonic activity sustained.

The hemispheric dichotomy in areal contractional strain is not easily explained for a one-plate planet thought to have experienced uniform radial contraction. It may suggest a generally thicker and/or stronger lithosphere in the northern hemisphere that has inhibited formation of more uniformly distributed, lithosphere-penetrating thrust faults. The dichotomy in distribution of smooth plains also has implications for the lithosphere of the northern hemisphere. The prevalence of wrinkle ridge-type deformation in smooth plains suggests stresses from subsidence and flexure due to basalt-like volcanic loads on a thinner or weaker lithosphere locally dominated stresses from global contraction. It has been shown on Earth that variations in effective elastic thickness *T*_*e*_ generally correlate with variations in crustal thickness *T*_*c*_—low values of *T*_*c*_ correspond to low values of *T*_*e*_^60^—and with the thickness of the seismogenic crust^61^. If this relationship holds for Mercury, smooth plains in the northern hemisphere might be expected to be underlain by relatively thin crust and correspondingly thin elastic lithosphere. This appears to be mostly the case with the northern smooth plains, the smooth plains of Suisei Planitia and Sobkou Planitia, and much of the Caloris basin underlain by relatively thin crust (see ref. ^26^, Fig. 10). Exceptions are much of the Caloris exterior smooth plains. One explanation for the apparent paradox of a generally thicker, stronger elastic lithosphere in the northern hemisphere and locally thinner, weaker lithosphere associated with the smooth plains is the effect of large impacts and/or the thermal effects related to flood volcanism. As is likely the case for the lunar nearside mare, deformation of smooth plains volcanic sequences by subsidence and flexure may have been facilitated by locally thinned or weakened lithosphere.

## Methods

Details about the methods used can be found in the manuscript and in the Supplementary Information.

## Supplementary information

Supplementary Information

## Data Availability

Data are available on the Smithsonian’s Figshare site (10.25573/data.12482453). The raw and calibrated image data that support the findings of this study are available from Planetary Data System Cartography and Imaging Sciences Node (https://pds-geosciences.wustl.edu/missions/messenger/index.htm).
