# Supplementary material for: A case for limited global contraction of Mercury
Source: Commun Earth Environ. 2021 Jan 14;2(1):9. doi: 10.1038/s43247-020-00076-5 (PMC7808997; doi:10.1038/s43247-020-00076-5)
Supplement: Supplementary file 1 — Supplementary Information [file 43247_2020_76_MOESM1_ESM.pdf]

A case for limited global contraction of Mercury

Thomas R. Watters[1]

1. Center for Earth and Planetary Studies, National Air and Space Museum, Smithsonian Institution, Washington, D.C. 20560-0315, USA.

Correspondence and requests for materials should be addressed to [1]T.R. Watters (e-mail: watterst@si.edu).

Supplementary Information

Supplementary Note 1, 2, 3; Supplementary Figures 1, 2; Supplementary Table 1; and Supplementary References.

The supplementary text and table provide further details on the approach to mapping the contractional tectonic landforms, the method used to determine the maximum relief of lobate thrust fault scarps and derive the fault displacement-fault length relations of the population, and the approach in previous studies to estimate global radius change.

Supplementary Note 1. Guiding Principles

In this study, each lobate scarp and high-relief ridge is interpreted to have a single primary thrust fault controlling the surface expression and contractional strain of the structure, and thus each is mapped with a single polyline. Lobate scarps are clearly distinguished by their asymmetric cross-section, consisting of a steeply sloping scarp face and a gently sloping back limb. The polyline is placed at the base of the vergent side of the scarp where there is often evidence of an offset and the thrust fault is interpreted to break the surface. High-relief ridges are characterized by a more symmetric form and generally exhibit no clear expression of surface breaking faults. The polyline is placed along the midline of the ridge since it is interpreted to involve a blind thrust fault. This criteria for identification is consistent with kinematic and mechanical models of these structures[1-7]. Wrinkle ridges are easily distinguishable from lobate scarps and high-relief ridges based on geologic setting, morphologic complexity, and relief. Wrinkle ridges occur exclusively in smooth plains volcanic sequences. They are morphologically complex structures that are often

composed of a number of superimposed landforms, usually consisting of a broad arch and superposed narrow, asymmetric ridges.

The dimensions of measured lobate scarps were determined using the best available topography in a given region (Table S1). Profiles across the lobate scarps were then generated in multiple locations to determine the maximum relief. Lengths of the scarps were determined from latitude and longitude endpoints of digitized segments that formed individual polylines. Segment lengths and orientations were calculated and lengths summed for each polyline. The tectonic features were identified and digitized directly from MESSENGER high-incidence and moderate incidence angle mosaics with a pixel scale of ~166 m.

Supplementary Note 2. Variations in Reported Radius Change from Planetary Contraction

An example of the impact of the difference in approach taken in the interpretation and mapping of tectonic landforms by Byrne et al. (8) and in this study on estimates of contractional strain and radius change is illustrated in Figure S1. Multiple polylines, each interpreted to be primary thrust faults, are assigned to Enterprise Rupes by Byrne et al. (8) (Fig. S1a). Numerous other putative faults are mapped radiating from Enterprise Rupes. Analysis of the morphometry of the scarp, the inferred displacement profile of the thrust fault, and elastic dislocation modeling of the thrust fault, all suggest a single, primary fault can account for Enterprise Rupes[7]. The likely existence of secondary thrust faults is acknowledged, but they are expected to be relatively small accommodation faults and not to significantly contribute to the contractional strain. Thus, Enterprise Rupes is represented by a single thrust fault (Fig. S1b). Another example contrasting the results of the difference in guiding principles is illustrated by the mapping of the Antoniadi Dorsa, a prominent high-relief ridge in the northern hemisphere (Fig. S2). Along the length of the

ridge, multiple faults are assigned by Byrne et al. (8), some on both flanks of the landform (Fig. S2a). Many other features, some without significant topographic expression (i.e., lineaments), are interpreted by Byrne et al. (8) to be shortening structures (Fig. S2a). In this study, Antoniadi Dorsa is represented by a single polyline along the midline of the ridge based on the interpretation that the landform is controlled by a single, blind thrust fault (Fig. S2b) (see Text S1). Assigning multiple faults to a single tectonic landform that are weighted equally in assessments of contractional strain, an "all-mapped-faults-equal" approach, will result in large overestimates of the regional and global contractional strain.

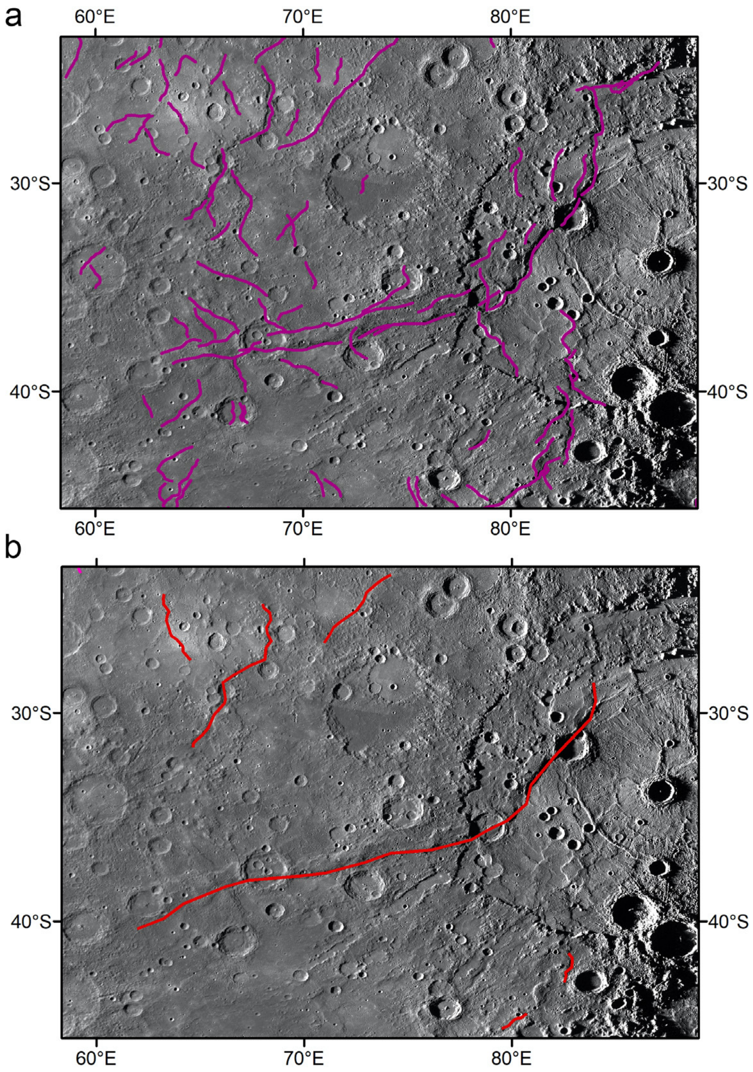

Supplementary Figure 1. Comparison of identification and mapping of Enterprise Rupes and other lobate scarps in the area using two different approaches to the interpretation of contractional tectonic landforms on Mercury. **a**) multiple polylines interpreted to be thrust faults (purple lines) associated with Enterprise Rupes by Byrne et al. (8). **b**) single polyline interpreted to be the primary thrust fault associated with Enterprise Rupes and other landforms interpreted to be lobate scarps (red lines). Each contractional landform is interpreted to be controlled by a single, primary thrust fault in this study.

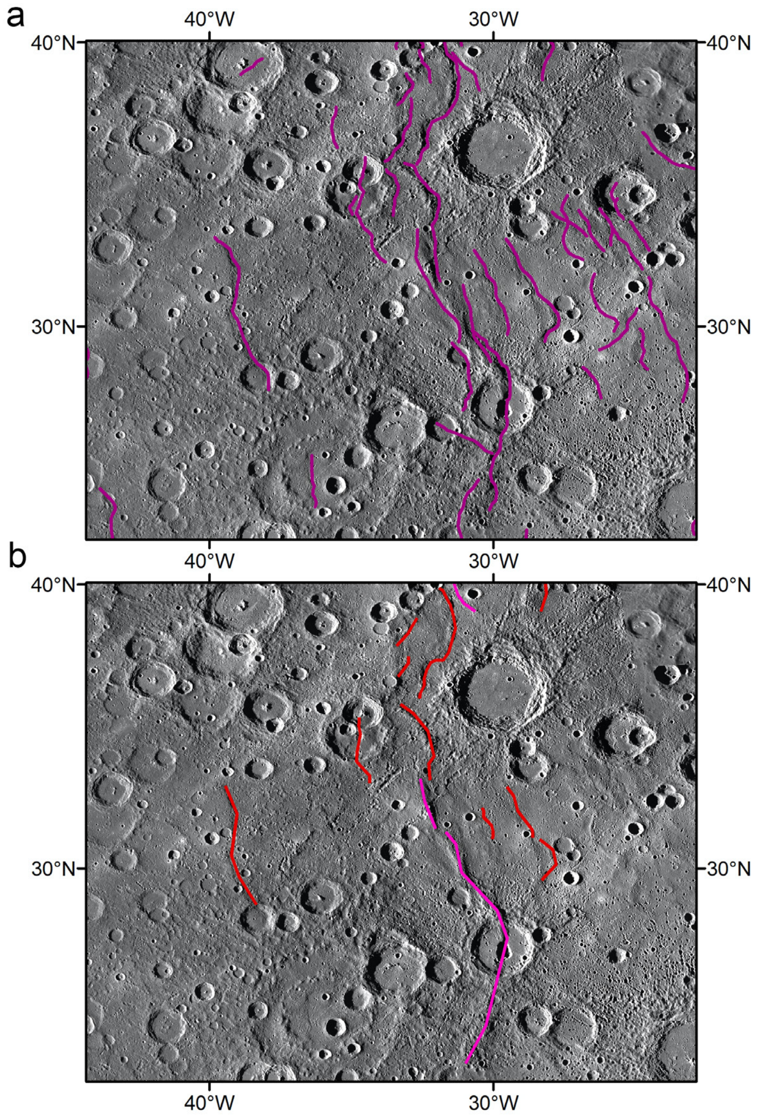

Supplementary Figure 2. Comparison of identification and mapping of the high-relief ridge Antoniadi Dorsa (lower right) and lobate scarps in the area using two different approaches to the interpretation of contractional tectonic landforms on Mercury. **a**) multiple polylines interpreted to be thrust faults associated with Antoniadi Dorsa and other features interpreted to be shortening structures (purple lines) by Byrne et al. (8). **b**) single polyline assigned to Antoniadi Dorsa (magenta lines) and to landforms interpreted to be lobate scarps (red lines). Each contractional landform is interpreted to be controlled by a single, primary thrust fault in this study.

Supplementary Note 3. Dimensions and Displacement-Length Relations of Lobate Thrust Fault Scarps

The dimensions of a representative sample of the population of lobate scarps were determined using the best available topography in a given region (Table S1). Topography was obtained from the Mercury Laser Altimeter (MLA)[9], from digital elevation models (DEMs) derived from stereo imaging[10, 11], and from a global DEM derived from image-based control-point network techniques[12]. Profiles across the lobate scarps were then generated in several locations near the length midpoint to determine the maximum relief. Lengths of the scarps were determined from latitude and longitude endpoints of digitized segments that formed individual polylines. Segment lengths were calculated and summed for each polyline.

Analysis of the displacement-length (*D/L*) relations of terrestrial faults shows that the maximum displacement D_{max} on a fault scales with the planimetric length of the fault[13-17], and this relation holds for planetary faults[3, 17-20]. A linear relationship $D_{max} = \gamma L$, where γ is a constant determined by rock type and tectonic setting[15], is supported by studies of faults in populations formed in uniform rock types[20, 21]. This scaling relationship has been shown to apply to all fault types (i.e., normal, strike-slip, and thrust) in a wide variety of tectonic settings and eight orders of magnitude in

length scale[14]. The amount of displacement on thrust faults is estimated using the measured maximum relief of the scarp h_{max}, generally measured at or near the length midpoint of the scarp, and the assumed fault plane dip θ. The maximum displacement necessary to restore the topography to a planar surface is given by $D_{max} = h_{max}/\sin\theta$ (19, 23). The assumed range of expected θ (25° to 35°) is consistent with faults associated with the Wind River thrust fault and thrust faults in the Rocky Mountain foreland of Wyoming, the best terrestrial analogs to planetary lobate scarps[5, 19]. Lower thrust fault dip angles (<25°) are not supported by forward mechanical modeling of lobate scarps on Mercury[7, 24, 25] and Mars[26].

Supplementary Table 1. Dimensions of Lobate Scarps

Lobate Scarp	Data Source	Lat (°)	Lon (°)	Minimum Elevation	Maximum Elevation	Relief (m)	Length (km)
Carnegie Rupes	MLA-GDR†	58.52	-53.25	-2058.375	-213.47	1844.905	278.83
Discovery Rupes	USGS-CPN§	-54.7	37.24	-764	-15.5	748.5	434.4
Enterprise Rupes	DLR‡ H14	-36.8	74.5	-2824	415	3239	985.32
Belgica Rupes	USGS-CPN	-50.4	63.3	-1762	-119	1643	374
Kuiper (unnamed)	DLR H6	-15.67	-46.41	159	1604	1445	286.3
Santa Maria Rupes	DLR H6	5.7	-19.8	1288	1939	651	222.5
Kuiper HRR (S_K4)	DLR H6	6	-18.29	1104	2899	1795	504.1
Debussy (unnamed)	USGS-CPN	-40.63	15.22	506.5	1160	653.5	306.3
Beagle Rupes	USGS-CPN	-2.3	101.2	378.5	1970	1591.5	612.63
Victoria Rupes	MLA-GDR	52.8	-34.3	-1190.46	-161.28	1029.18	396.9
Enheduanna (unnamed)	USGS-CPN	44.9	-31.78	33.5	1108.5	1075	204.02
Resolution Rupes	USGS-CPN	-63.25	-50.66	327	982.5	655.5	125
Adventure Rupes	USGS-CPN	-65.48	-65.3	-648.5	524.5	1173	302.6
Astrolabe Rupes	USGS-CPN	-42.55	-70.9	81	1024.5	943.5	248.8
Fram Rupes	USGS-CPN	-57.68	-93.2	-1265	71.5	1336.5	187.6
Hero Rupes	USGS-CPN	-57.68	-171.7	584	1675	1091	318.4
Unity Rupes	DLR H5	27.16	-84.8	-634	1310	1944	288.8
Calypso Rupes	USGS-CPN	19.53	43.52	-928	1074.5	2002.5	445.8
Nautilus Rupes	DLR H3	-27.8	67.24	-1772	-144	1628	361.4
Vostok Rupes	USGS-CPN	-36.9	-20.08	1328.5	2147.5	819	210.63
Zarya Rupes	USGS-CPN	-42.73	-20.39	-183.5	1100	1283.5	159.1
Eltanin Rupes	USGS-CPN	-73.23	96.61	-2651	-1699.5	951.5	257
Terror Rupes	USGS-CPN	-72.04	84.3	-2291.5	-1697	594.5	228.1
Palmer Rupes	USGS-CPN	-26.07	-106.28	-901.5	261	1162.5	278.6
Mirni Rupes	USGS-CPN	-38.3	-39.4	339	1272	933	279.3
Simonides (unnamed)	USGS-CPN	-29.1	-44.9	540.5	2032	1491.5	345
Gjoa Rupes	USGS-CPN	-65.3	-164.67	-1050.5	-112.5	938	308.8
Sinan (unnamed)	DLR H6	17.46	-30.68	1050	1743	693	178.6
Abu (unnamed)	DLR H6	17.5	-26.55	1065	1633	568	161.8
Donne B (unnamed)	DLR H6	0.98	-7.83	1999	3180	1181	271.8
Glinka (unnamed)	DLR H7	12.8	-112.5	-548	232	780	214.3

†MLA-GDR is gridded topography data obtained from the Mercury Laser Altimeter[9], ‡DLR are DEMs for Mercury quadrangles H5, H6, and H7 derived from stereo images obtained by the Mercury Dual Imaging System (MDIS) using photogrammetric analysis[10,11], §USGS-CPN is a global DEM generated from control-point network using MDIS images[12]. Unnamed lobate scarps are given unofficial names based on their proximity to named impact craters.

Supplementary References

1. Strom, R. G., Trask, N. J. & Guest J. E. Tectonism and volcanism on Mercury, *J. Geophys. Res.* **80**, 2478–2507 (1975).

2. Melosh, H. J. & McKinnon, W. B. in *Mercury* (eds Vilas, F., Chapman, C. R. & Matthews, M. S.) 374-400 (Univ. Arizona Press, 1988).

3. Watters, T. R., Robinson, M. S. & Cook, A. C. Topography of lobate scarps on Mercury: New constraints on the planet's contraction. *Geology* **26**, 991-994 (1998).

4. Watters, T. R., Robinson, M. S. & Cook, A. C. Large-scale lobate scarps in the southern hemisphere of Mercury. *Planet. Space Sci.* **49**, 1523-1530 (2001).

5. Watters, T. R., Schultz, R. A., Robinson, M. S. & Cook A. C. The mechanical and thermal structure of Mercury's early lithosphere. *Geophys. Res. Letts.* **29**, doi:10.1029/2001GL014308 (2002).

6. Watters, T. R., Robinson, M. S., Bina, C. R. & Spudis, P. D. Thrust faults and the global contraction Mercury, *Geophys. Res. Lett*. **31**, L04701, 10.1029/2003GL019171 (2004).

7. Watters, T. R., Montési, L.G.J., Oberst, J. & Preusker, F. Fault-bound valley associated with the Rembrandt Basin on Mercury, *Geophys. Res. Lett.* **43**, 11,536–11,544, doi:10.1002/2016GL070205 (2016).

8. Byrne, P. K. et al. Mercury: Global tectonics on a contracting planet. *Nat. Geosci.* **7**, 301-307 (2014).

9. Zuber M. T. et al. Topography of the northern hemisphere of Mercury from MESSENGER Laser Altimetry. *Science* **336**, 217-220 (2012).

10. Oberst, J., et al. The morphology of Mercury's Caloris basin as seen in MESSENGER stereo topographic models. *Icarus* **209**, 230-238 (2010).

11. Preusker F. et al. Toward High-Resolution Global Topography of Mercury from MESSENGER Orbital Stereo Imaging: A Prototype Model for the H6 (Kuiper) Quadrangle. *Planetary and Space Science* **142**, 26-37, doi:10.1016/j.pss.2017.04.012 (2017).

12. Becker, K.J. et al. First global digital elevation model of Mercury (Abstract), *LPSC 47*, #2959 (2016).

13. Walsh, J. & Watterson, J. Analysis of the relationship between displacements and dimensions of faults. *J. Struct. Geol*. **10**, 239-247 (1988).

14. Cowie, P. A. & Scholz, C. H. Physical explanation for the displacement-length relationship of faults using a post-yield fracture-mechanics model. *J. Struct. Geol.* **14**, 1133-1148 (1992a).

15. Cowie, P. A. & Scholz, C. H. Displacement-length scaling relationship for faults data synthesis and discussion. *J. Struct. Geol.* **14**, 1149-1156 (1992b).

16. Gillespie, P. A., Walsh, J. J. &Watterson, J. Limitations of dimension and displacement data from single faults and the consequences for data analysis and interpretation. *J. Struct. Geol.* **14**, 1157-1172 (1992).

17. Schultz, R. A. & Fori, A. N. Fault-length statistics and implications of graben sets at Candor Mensa, Mars. *J. Struct. Geol.* **18**, 272-383 (1996).

18. Schultz, R. A. Displacement-length scaling for terrestrial and Martian faults: Implications for Valles Marineris and shallow planetary grabens. *J. Geophys. Res.* **102***,* 12009-12015 (1997).

19. Watters, T. R., Schultz, R. A. & Robinson, M. S. Displacement-length relations of thrust faults associated with lobate scarps on mercury and Mars: Comparison with terrestrial faults. *Geophys. Res. Lett.* **27**, 3659–3662 (2000).

20. Schultz, R. A., Soliva, R., Okubo, C. H. & Mége, D. in *Planetary Tectonics* (eds Watters, T. R. & Schultz, R. A.) 457–510 (Cambridge Univ. Press, New York, 2010).

21. Dawers, N. H., Anders, M. H., & Scholz, C. H. Growth of normal faults: displacement length scaling. *Geology* **21**, 1107-1110 (1993).

22. Clark, R. & Cox, S. A modern regression approach to determining fault displacement-length scaling relationships. *J. Struct. Geol*. **18**, 147-154 (1996).

23. Wojtal, S. F. Changes in fault displacement populations correlated to linkage between faults. *J. Struct. Geol.* **18**, 265-279 (1996).

24. Watters, T. R., Schultz, R. A., Robinson, M. S. & Cook A. C. The mechanical and thermal structure of Mercury's early lithosphere. *Geophys. Res. Letts.* **29**, doi:10.1029/2001GL014308 (2002).

25. Egea-Gonzalez, I., et al. Depth of faulting and ancient heat flows in the Kuiper region of Mercury from lobate scarp topography. *Planet. Space Sci.* **60**, 193–198 (2012).

26. Schultz, R.A., Watters, T.R. (2001) Forward mechanical modeling of the Amenthes Rupes thrust fault on Mars, *Geophys. Res. Lett., 28*, 4659–4662.
